# Human Immune Response to Influenza Neuraminidase After Vaccination: A Systematic Review

**DOI:** 10.1111/irv.70192

**Published:** 2025-12-09

**Authors:** Vardhini Ganesh, Justin Iampietro, Saranya Sridhar, Ana P. Goncalvez

**Affiliations:** ^1^ Sanofi R&D Vaccines Waltham Massachusetts USA; ^2^ Sanofi Vaccines Reading UK

**Keywords:** immunogenicity, influenza, neuraminidase, neutralizing antibodies, seasonal vaccine

## Abstract

**Background:**

Licensed influenza vaccines primarily target hemagglutinin (HA)‐related immunity, but neuraminidase (NA)‐based immunity is gaining attention as an independent correlate of protection. Inactivated influenza vaccines contain unspecified quantities of residual NA, with limited understanding of the resulting antibody induction and durability.

**Methods:**

We conducted a systematic literature review across PubMed, Embase, Cochrane Library, ClinicalTrials.gov, and Trialtrove to identify studies reporting NA‐related immunity following influenza vaccination. Primary outcomes included pre‐existing and post‐vaccination NA‐inhibiting (NAI) antibody titers.

**Results:**

Analysis of 40 articles, predominantly examining split‐virus vaccines, revealed NAI antibody titers increased 5–7‐fold at days 8–30 post‐vaccination compared to baseline across all three NA (sub)types. NAI antibody geometric mean titers showed greater variability against N1 and N2 than against influenza B virus NAs. No significant differences in pre‐vaccination and post‐vaccination NAI antibody titers were observed between adults (18–64 years) and the elderly (≥ 65 years) for the N1 subtype. However, statistical differences were observed post‐vaccination (days 8–30) between these age groups for N2 (*p* = 0.04) and NB (*p* = 0.01) (sub)types. NAI antibody responses remained durable, persisting at 2–16‐fold above pre‐vaccination levels through days 91–180.

**Conclusion:**

Current influenza vaccines induce durable NAI responses in adults for up to 6 months. These findings provide valuable insights into pre‐vaccination and post‐vaccination responses to residual NA, which can guide future vaccine development strategies. Enhanced understanding of NA immunogenicity should inform public health approaches to improve global influenza prevention and control.

## 1 Introduction

Influenza is an infectious respiratory disease, caused predominantly by influenza A and B viruses, with seasonal transmission surges leading to regular epidemics [1]. It is estimated that 290,000–650,000 respiratory deaths annually are caused by influenza [2], and it imposes a substantial economic burden [3].

Developing vaccines that provide high levels of protection from influenza is an annual challenge. Error‐prone viral ribonucleic acid (RNA) replication leads to the accumulation of advantageous mutations in viral proteins, often leading to a mismatch between the antigenic epitopes of circulating strains and any immunity built from previous infections or vaccinations (antigenic drift) [1, 4]. Hemagglutinin (HA) and neuraminidase (NA) are influenza surface glycoproteins that undergo such mutations. HA mediates viral entry and presently represents the main antigenic component of licensed influenza vaccines [1]. However, NA, which supports the budding and release of new virions following replication, is also antigenic and capable of stimulating host immune responses [1, 5].

Approved influenza vaccines are standardized based on their antigenic HA content [6], and their effectiveness is determined by the surrogate marker of hemagglutination‐inhibiting (HAI) antibodies in the blood: a HAI titer of 1:40 is accepted as a correlate of protection following vaccination [6, 7].

Currently, attention is increasingly turning towards the inclusion of NA‐based immunity to broaden protection against influenza [7, 8]. In natural infection, NA can elicit strong humoral immune responses that have the potential to surpass those raised against HA epitopes [8]. Anti‐NA antibodies have been shown to have broad specificity across historical strains of influenza A [8]; there is evidence to suggest that genetic changes in NA occur more slowly than changes in HA, leading to a degree of NA epitope conservation between strains [9]. Anti‐NA antibodies have demonstrated robust inhibition of NA's enzymatic activity, preventing viral release from infected cells and stimulating antibody‐dependent cell‐mediated and complement‐dependent cytotoxicities to support the clearance of influenza infections as well as the attenuation of symptoms' severity and duration [8, 10]. Moreover, studies suggest that NAI antibody titers are independent correlates of protection and may serve as a stronger predictor of protection than HAI titers [11]. The development of new influenza vaccines that include NA necessitates an understanding of anti‐NA immunity in humans [12, 13]. Current approved vaccines, particularly split vaccines, contain NA, although this is not routinely quantified. Despite the presence of NA in current vaccines and the potential impact of anti‐NA antibodies on protection, few reports focus on anti‐NA immune responses following vaccination.

Here, we present a systematic literature review of the NA immunogenicity of influenza vaccines in humans. To the authors' knowledge, this is the first systematic review to assess vaccination‐induced NA immunogenicity aiming to fill a critical knowledge gap regarding baseline NA responses and the range of responses following vaccination. Understanding these patterns is crucial for informing clinical studies across different age groups and with various commercially available vaccines, as comprehensive data on functional NA antibodies elicited by these vaccines remain limited. Our findings should provide valuable insights to advance the development of more effective NA‐containing influenza vaccines.

## 2 Methods

### 2.1 Search Strategy and Study Selection

This systematic review was conducted in accordance with the Preferred Reporting Items for Systematic Review and Meta‐Analyses (PRISMA) guidelines [14]. Study selection criteria were established using the Population, Intervention, Comparison and Outcome (PICO) framework [15].

We evaluated clinical trials and observational studies reporting neuraminidase inhibition (NAI) antibody titers. For population criteria, studies were required to define participant age, enabling stratification into subgroups (0–5, 6–18, 18–64, and ≥ 65 years). No restrictions were imposed regarding study setting or participant characteristics, including vaccination history or comorbidities. Exclusion criteria encompassed studies lacking NA immunogenicity data, uncontrolled trials, investigations utilizing NA inhibitor drugs, influenza type C‐related research, and therapeutic vaccine outcome studies.

Interventions comprised all influenza vaccine types and valencies (inactivated whole virus, split virus, subunit influenza antigen vaccines, LAIVs, RIVs, with or without adjuvant). Messenger RNA (mRNA)‐based vaccines were excluded as no NA‐targeting vaccines of this type existed at review time. Studies were included irrespective of comparator, with placebo group data incorporated where available.

Primary endpoints comprised neuraminidase (NA) antibody responses measured via enzyme‐linked lectin assay (ELLA) and enzyme‐linked immunosorbent assay (ELISA). Specific measures included geometric mean titers (GMTs), seroprotection rates (defined as the proportion of subjects with NAI titers > 1:10 and/or > 1:40), seroconversion rates (assessed by fold rise in NAI titers), and breadth of immunological response across multiple temporal assessments.

The literature search employed MeSH and free text terms (Tables S1–S5) across PubMed, Embase, Cochrane Library, ClinicalTrials.gov, and Trialtrove (1970–2024). Following duplicate removal, two authors (VG and JI) independently screened records using DistillerSR (Evidence Partners, Ottawa, Canada) with a standardized scoring method (1 = *definitely exclude*, 5 = *maybe include*, 10 = *definitely include*). Full‐text screening was subsequently performed against predetermined eligibility criteria. Discrepancies were resolved by independent assessment from another author (SS). Non‐English publications were excluded.

### 2.2 Data Extraction

Data from eligible publications were extracted to Microsoft Excel by two authors (VG and JI). Data were collected as presented in the publications; where data were only presented graphically, numerical estimates were not made to facilitate extraction. Where necessary, the corresponding authors were contacted to request further data or clarification. Outcomes related to NA antibody GMTs, NA seroconversion, and fold rise in NA antibodies were collected for each influenza strain. An independent reviewer verified the data extracted from the original source publications.

### 2.3 Risk of Bias Assessment and Statistical Analysis

Risk of bias assessment was conducted using risk of bias 2 (RoB 2) [16] for randomized studies and the “risk of bias in nonrandomized studies ‐ of interventions” (ROBINS‐I) [17] for observational/nonrandomized studies. Two authors (VG and JI) independently evaluated all included studies, comparing scores and justifications, with discrepancies resolved by a third author (APG) according to established guidelines. Risk bias assessments were visualized using the risk‐of‐bias visualization (robvis) package from R [18].

Data synthesis, calculations, and visualization were performed using Tableau Desktop v2020.3. Following metadata nomenclature harmonization across studies, comprehensive data synthesis calculations generated a central dataset enabling cross‐trial analyses of two primary endpoints: geometric mean titers (GMTs) and fold rises. These were analyzed by influenza neuraminidase (NA) (sub)types N1, N2, and B across four time periods: time (T)0 = day (D)0; T2 = D8–30; T3 = D31–90; and T4 = D91–180.

For statistical analysis, GMT values were log2‐transformed to harmonize data across datasets. Where GMT confidence intervals were reported, sample standard deviations were calculated from log2‐transformed values, followed by anti‐log transformation to compute original‐scale GMTs. Subgroup analyses were stratified by NA (sub)type. Data distribution was represented through box‐and‐whisker plots with reference lines, where boxes indicated the middle 50% of data (middle two quartiles) with whiskers displaying all points within 1.5 times the interquartile range, overlaid with individual study data points.

While the broad scope precluded meta‐analysis, neuraminidase inhibition (NAI) immunogenicity responses between age groups were compared using box‐and‐whisker plots and one‐way ANOVA testing.

## 3 Results

### 3.1 Study Selection and Characteristics

**FIGURE 1:**
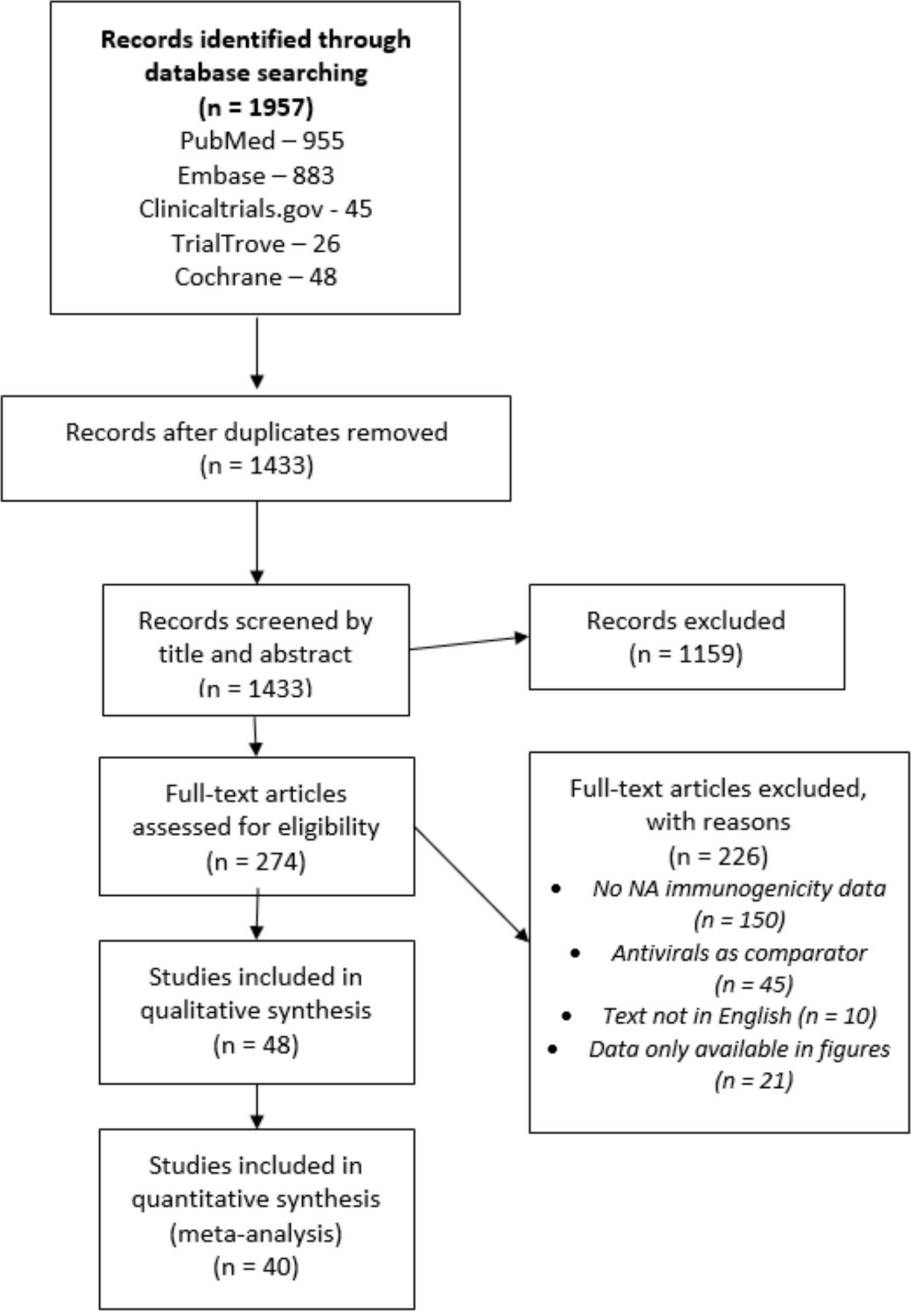
PRISMA flowchart summarizing the literature search.

The systematic search yielded 1957 publications, with 1433 remaining after duplicate removal (Figure 1). Following initial screening of titles and abstracts, 274 full‐text articles were assessed for eligibility. This evaluation resulted in 48 studies meeting the inclusion criteria for qualitative analysis. Of these, 40 studies fulfilled the requirements for quantitative analysis and were subsequently utilized for inferential statistical assessments in this investigation. These 40 studies formed the core dataset for our comprehensive analysis of neuraminidase inhibition antibody responses following influenza vaccination. Study characteristics are summarized in Table 1. Geographic distribution of studies included the United States (*n* = 18), Poland (*n* = 10), United Kingdom (*n* = 5), with additional studies from various European and Asian countries, Australia (*n* = 2), and Chile (*n* = 1).

**TABLE 1:** Characteristics of included studies (*n* = 48).

Study characteristics	Number of studies	List of references
**Age category**		
Adults (aged 18–64 years)	30	[11, 19, 20, 21, 22, 23, 24, 25, 26, 27, 28, 29, 30, 31, 32, 33, 34, 35, 36, 37, 38, 39, 40, 41, 42, 43, 44, 45, 46, 47]
Elderly (aged ≥ 65 years)	15	[21, 24, 30, 31, 36, 38, 40, 41, 46, 47, 48, 49, 50, 51, 52]
Children	15	[40, 41, 53, 54, 55, 56, 57, 58, 59, 60, 61, 62, 63, 64, 65]
**Method**		
Standard ELLA	21	[11, 19, 20, 21, 22, 31, 35, 39, 40, 42, 46, 47, 48, 49, 52, 53, 54, 60, 61, 64, 65]
Other NAI assays	27	[23, 24, 25, 26, 27, 28, 29, 30, 32, 33, 34, 36, 37, 38, 41, 43, 44, 45, 50, 51, 55, 56, 57, 58, 59, 62, 63]
ELISA	3	[26, 36, 40]
**Intervention (vaccine type)**		
Split virus	36	[11, 19, 20, 21, 22, 23, 24, 25, 26, 27, 28, 29, 30, 31, 32, 33, 34, 35, 36, 37, 38, 41, 48, 49, 50, 51, 52, 53, 54, 55, 56, 57, 58, 59, 60, 61]
LAIV	8	[11, 19, 20, 29, 35, 42, 60, 61]
Subunit	11	[26, 27, 29, 38, 39, 40, 43, 45, 62, 63, 64]
Whole virus inactivated	7	[27, 29, 34, 44, 47, 63, 65]
Experimental virus	1	[46]
**Intervention (valency)**		
Monovalent	16	[22, 26, 29, 30, 32, 34, 44, 46, 47, 54, 56, 57, 58, 59, 63, 65]
Bivalent	2	[28, 29]
Trivalent	28	[11, 19, 20, 21, 23, 24, 25, 26, 27, 29, 31, 33, 35, 36, 37, 38, 39, 40, 41, 42, 43, 45, 48, 49, 50, 51, 52, 62]
Quadrivalent	6	[48, 53, 55, 60, 61, 64]
**Adjuvant**		
Studies with adjuvant	8	[22, 27, 28, 29, 43, 50, 54, 63]
Studies without adjuvant	40	[11, 19, 20, 21, 23, 24, 25, 26, 30, 31, 32, 33, 34, 35, 36, 37, 38, 39, 40, 41, 42, 44, 45, 46, 47, 48, 49, 51, 52, 53, 55, 56, 57, 58, 59, 60, 61, 62, 64, 65]
**Number of doses of vaccine**		
One	35	[11, 19, 21, 23, 24, 25, 26, 28, 29, 30, 31, 33, 34, 35, 36, 38, 39, 40, 41, 43, 45, 48, 49, 50, 51, 52, 53, 55, 56, 57, 58, 59, 60, 61, 62]
More than one	16	[20, 22, 27, 29, 32, 37, 42, 44, 46, 47, 53, 54, 60, 66, 67, 68]
**Neuraminidase (sub)type**		
Only N1	11	[30, 37, 44, 46, 47, 49, 54, 60, 61, 63, 65]
Only N2	10	[11, 26, 34, 35, 43, 50, 56, 57, 58, 59]
Only B	0	—
More than one NA subtype (N1 & N2; N1 & N2 & B)	26	[19, 20, 21, 23, 24, 25, 27, 28, 29, 31, 32, 33, 36, 38, 39, 40, 41, 42, 45, 48, 51, 52, 53, 55, 62, 64]
N9	1	[22]
**Administration route**		
Intramuscular (IM)	41	[11, 19, 20, 21, 22, 23, 24, 26, 27, 28, 29, 30, 31, 32, 33, 35, 36, 37, 38, 39, 40, 41, 43, 44, 45, 46, 47, 48, 49, 50, 51, 52, 53, 54, 55, 56, 57, 58, 59, 64, 65]
Intranasal (IN)	8	[19, 20, 29, 35, 41, 42, 60, 61]
Subcutaneous (SC)	5	[25, 29, 34, 62, 63]
**Subject health status**		
No condition	41	[11, 19, 20, 22, 23, 24, 26, 27, 28, 29, 30, 31, 32, 33, 34, 35, 36, 37, 39, 40, 41, 42, 43, 44, 45, 46, 48, 49, 50, 52, 53, 54, 56, 57, 58, 59, 60, 61, 63, 64, 65]
One or more health conditions	8	[21, 25, 33, 38, 47, 51, 55, 62]

Abbreviations: ELISA, enzyme‐linked immunosorbent assay; ELLA, enzyme‐linked lectin assay; IM, intramuscular; IN, intranasal; LAIV, live‐attenuated influenza vaccine; SC, subcutaneous.

While both enzyme‐linked lectin assay (ELLA) and enzyme‐linked immunosorbent assay (ELISA) immunogenicity assessments were included in screening criteria, three ELISA‐based studies were excluded due to inter‐laboratory methodological variations. The analysis identified 35 studies investigating split‐virus vaccines, 8 examining live‐attenuated influenza vaccines (LAIVs), 11 evaluating subunit vaccines, and 7 assessing whole virus inactivated vaccines. Quantitative analysis focused predominantly on split‐virus vaccines, which constituted the largest and most robust dataset [11, 19, 20, 21, 22, 23, 24, 25, 26, 27, 28, 29, 30, 31, 32, 33, 34, 35, 36, 37, 38, 48, 49, 50, 51, 52, 53, 54, 55, 56, 57, 58, 59, 60, 61]. Limited publication numbers for LAIV (*n* = 8) and whole‐virus vaccines (*n* = 7) precluded comprehensive quantitative analysis across designated timepoints. Of the 40 included articles, 25 were subjected to quantitative analysis focusing on split‐virus vaccines, as detailed in subsequent sections. These included 10 randomized studies [11, 19, 20, 26, 35, 36, 39, 48, 49, 50] and 15 observational/nonrandomized studies [21, 23, 24, 27, 28, 29, 31, 37, 38, 51, 52, 53, 55, 60, 61].

### 3.2 Risk of Bias Within Studies That Focused on Split‐Virus Vaccines

The risk of bias assessments for the studies included in the analysis are summarized in Figure S1A for randomized controlled trials (RoB2 tool) and Figure S1B for nonrandomized controlled trials (ROBINS‐I tool). The outcomes reported in two out of 10 randomized controlled trials had an overall high risk of bias and those for three out of 15 nonrandomized controlled trials had an overall critical risk of bias. The overall judgments for the remainder of the studies were either some concerns/low risk of bias or moderate/low risk of bias, respectively.

### 3.3 Early NAI Antibody Responses Against Homologous NAs

**FIGURE 2:**
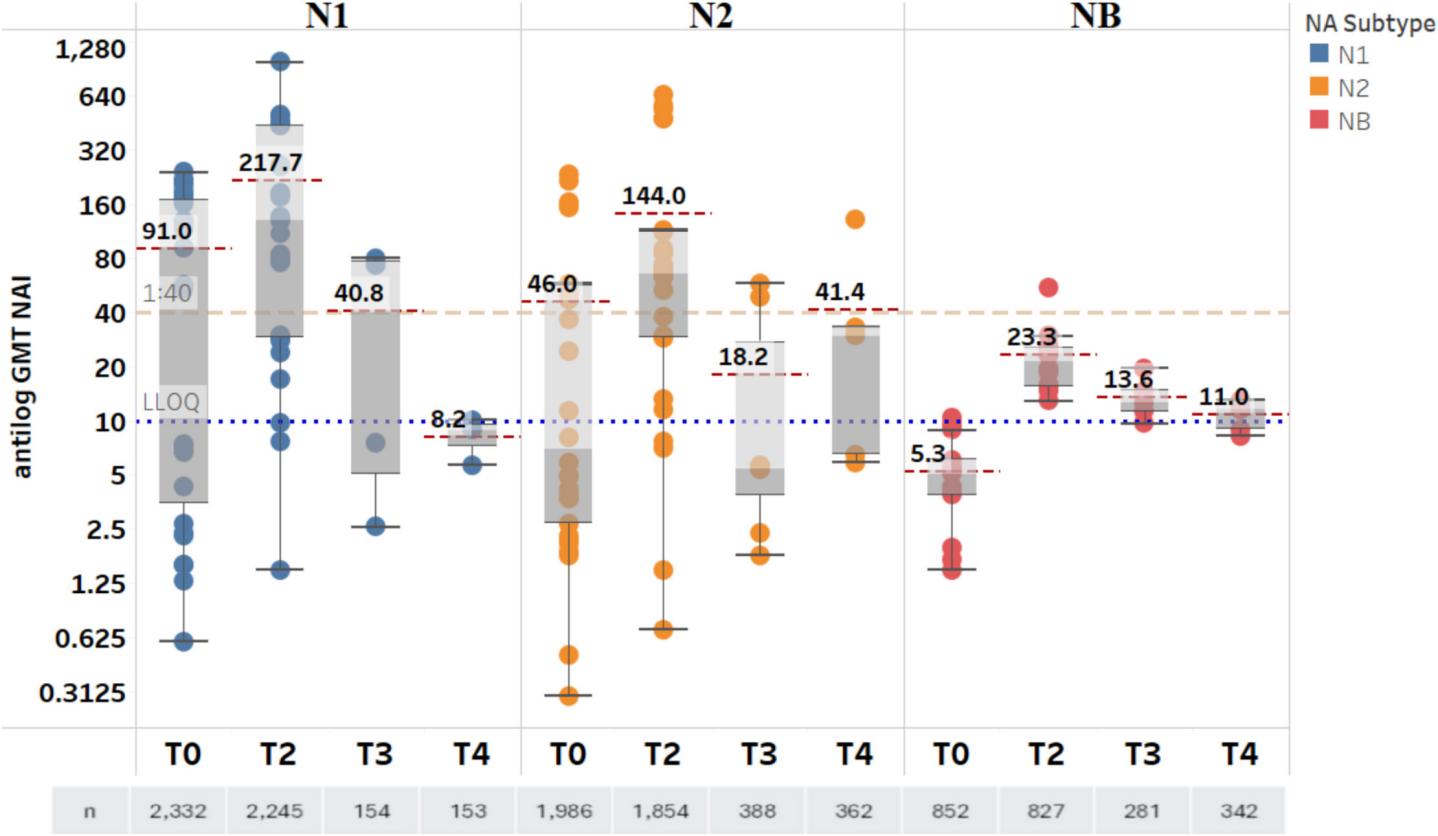
NAI antibody GMTs against homologous NA subtypes for split‐virus vaccines. Data are summarized as box‐and‐whisker plots, where the boxes indicate the middle 50% of the data (i.e., middle two quartiles of the data's distribution) with whiskers to display all points within 1.5 times the interquartile range, and overlayed with the individual study data points included (each dot color indicates NA subtype). The bold numbers for each pane indicate the aggregated GMTs (red dashed line) for a particular time point within each NA (sub)type. The dashed orange line indicates 1:40 titer (assumed seroprotection level) and the dotted purple line indicates 1:10 titer (LLOQ). T0 = D0; T2 = D8–30; T3 = D31–90; T4 = D91–180. D, day; GMT, geometric mean titer; LLOQ, lower limit of quantification; NA, neuraminidase; NAI, neuraminidase inhibition; T, time point.

**FIGURE 3:**
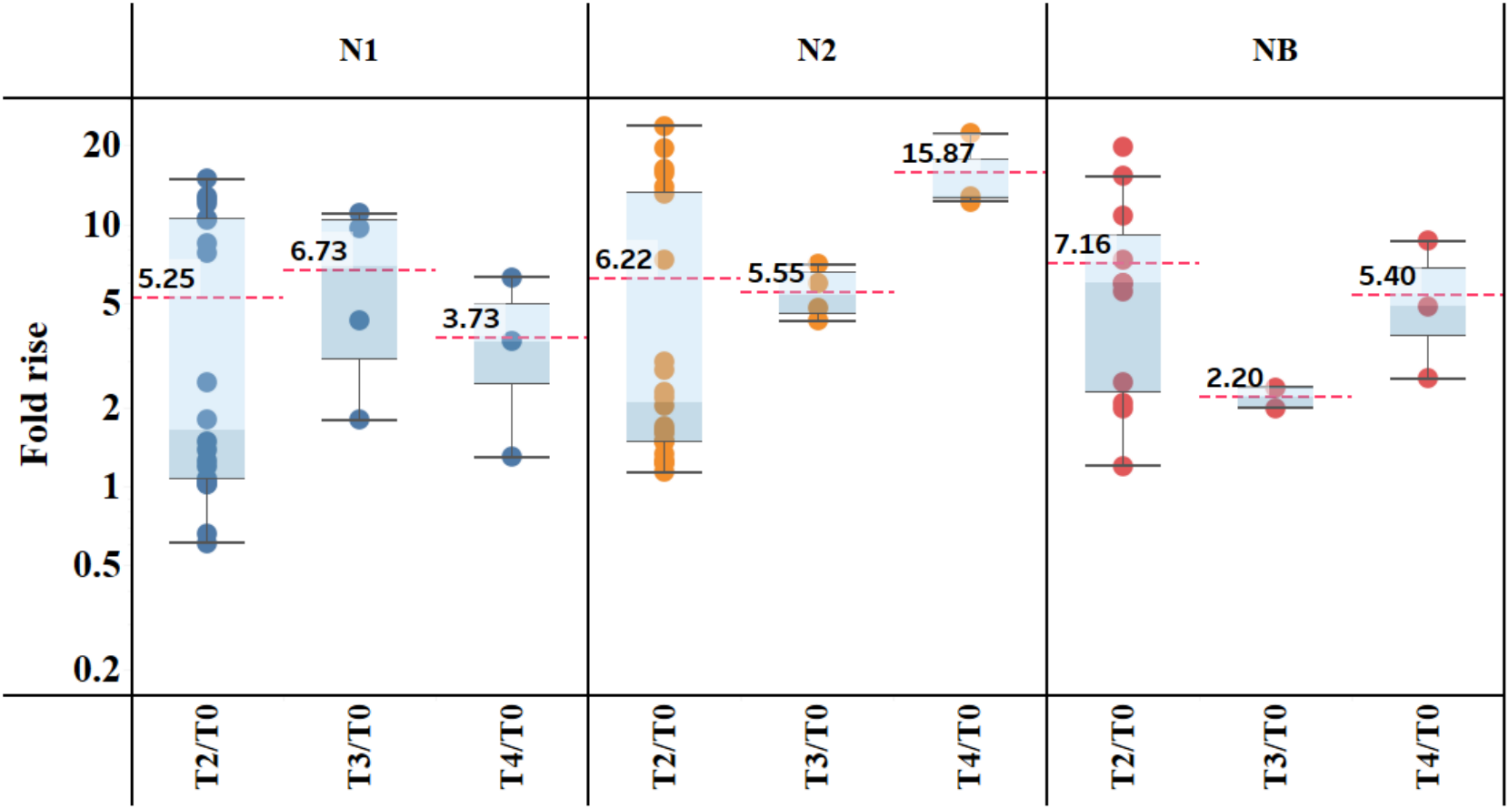
Fold rise from pre‐vaccination of NAI antibody GMTs against homologous NA subtypes for split‐virus vaccines (all types). Data are summarized as box‐and‐whisker plots, where the boxes indicate the middle 50% of the data (i.e., middle two quartiles of the data's distribution) with whiskers to display all points within 1.5 times the interquartile range, and overlayed with the individual study data points included (each dot indicates unique data from the publications). The bold numbers for each pane indicate the aggregated GMT fold‐rise for a particular time point within each NA (sub)type. T0 = D0; T2 = D8–30; T3 = D31–90; T4 = D91–180. D, day; GMT, geometric mean titer; NA, neuraminidase; NAI, neuraminidase inhibition; T, time point.

The NAI antibody GMTs to homologous (vaccine‐matched) influenza NA subtypes N1 and N2, and influenza B virus NAs pre‐ and post‐vaccination with split‐virus influenza vaccines are summarized in Figure 2. Baseline aggregated GMTs against N1 (91 [1/dil]) and N2 (46 [1/dil]) were much higher than those observed against influenza B virus NAs (5.3 [1/dil]). Post‐vaccination NAI antibody titers at T2 (D8–30 post‐vaccination) were boosted compared to baseline for all influenza NAs (aggregated GMTs at T2: N1 = 217.7 [1/dil], N2 = 144 [1/dil]; influenza B virus NAs = 23.2 [1/dil]), but there was greater GMT variability against N1 and N2 than that observed for influenza B virus NAs (Figure 2). The fold rise in NAI antibody GMTs against homologous NA (sub)types from pre‐vaccination (T0) to post‐vaccination is summarized in Figure 3. A 5–7‐fold increase was seen in NAI antibody GMTs against all three NA (sub)types from pre‐vaccination level to T2 (D8–30 post‐vaccination).

**FIGURE 4:**
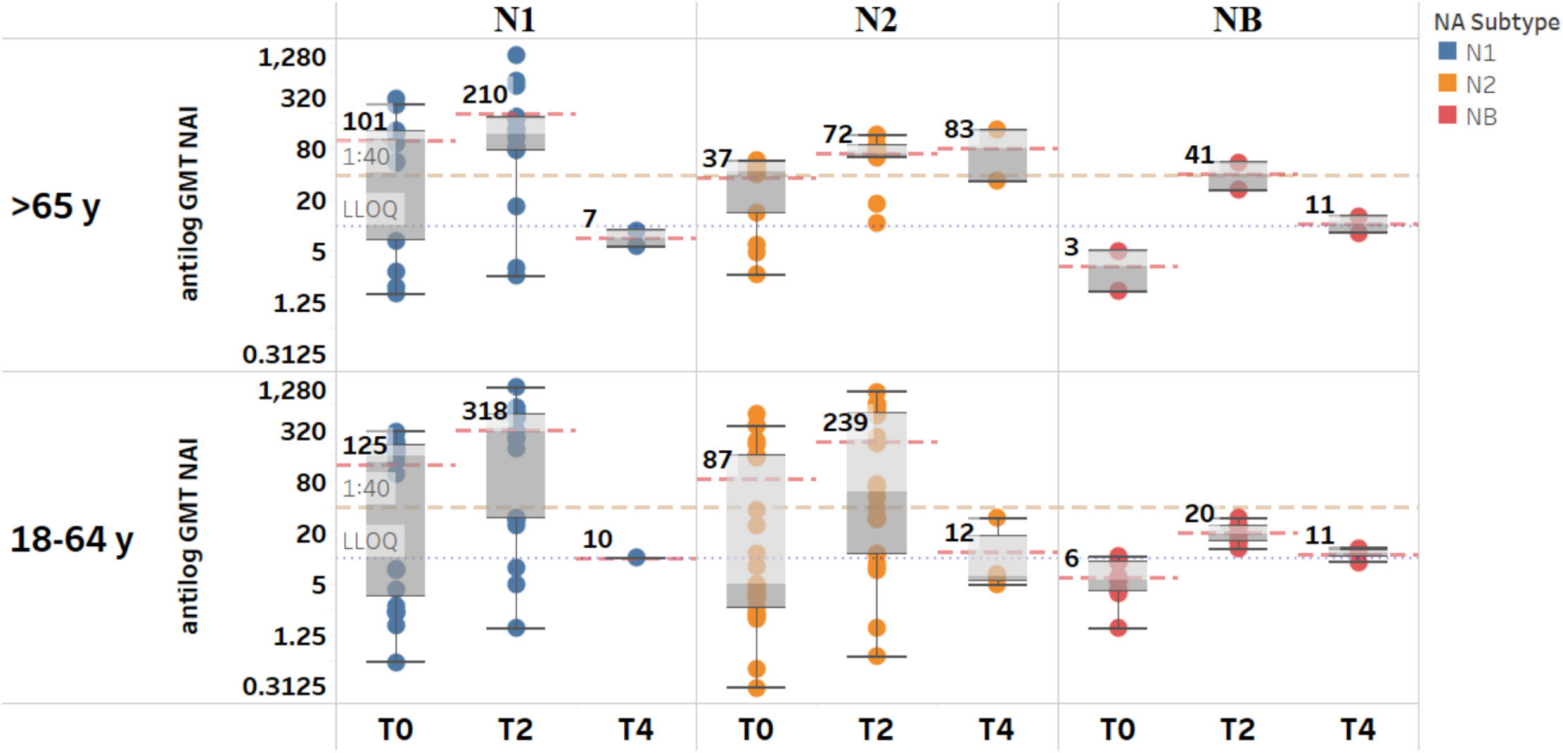
NAI antibody GMTs against homologous NA subtypes in adults (18–64 years) and the elderly (≥ 65 years) (split‐virus vaccines). Data are summarized as box‐and‐whisker plots, where the boxes indicate the middle 50% of the data (i.e., middle two quartiles of the data's distribution) with whiskers to display all points within 1.5 times the interquartile range, and overlayed with the individual study data points included (each dot color NA subtype). The bold numbers for each pane indicate the aggregated GMTs (dashed red line) for a particular time point within each NA (sub)type. The dashed orange line indicates 1:40 titer (assumed seroprotection level) and the dotted purple line indicates 1:10 titer (LLOQ). T0 = D0; T2 = D8–30; T4 = D91–180. D, day; GMT, geometric mean titer; LLOQ, lower limit of quantification; NA, neuraminidase; NAI, neuraminidase inhibition; T, time point, y, years.

There were a limited number of studies in older adults (≥ 65 years) (*n* = 8) with split vaccines and only two studies assessed responses to influenza B virus NAs in this age group. Our analysis revealed important age‐related patterns in neuraminidase inhibition (NAI) antibody responses. For the N1 subtype, no significant differences were observed between young adults (18–64 years) and the older adults (≥ 65 years) at any timepoint (*p* > 0.05). In contrast, young adults generated significantly higher NAI antibody titers against the N2 (*p* = 0.04) and NB subtypes (*p* = 0.01) during early post‐vaccination (days 8–30), as shown in Figure 4. These findings suggest subtype‐specific immunosenescence effects that may inform future age‐targeted vaccine development strategies.

### 3.4 Durability of NAI Antibody Responses Against Homologous NAs

Studies reporting antibody responses beyond D30 showed antibody GMTs remained about 2–16‐fold higher than those observed pre‐vaccination in the few studies that had data at these later time points (Figure 3). Although the fold rise for the N2 subtype at T4 (D91–180 post‐vaccination) appeared higher than that at T2 (D8–30 post‐vaccination), the number of publications assessing this was limited, and the assessment was based on different studies between the two time points. In general, the data suggest some waning of NAI titers for the later time points (T3 [D31–90 post‐vaccination] and T4 [D91–180 post‐vaccination]) compared to those at T2 (D8–30 post‐vaccination) (Figure 2).

### 3.5 Post‐Vaccination Responses to Various Commercial Vaccines

**FIGURE 5:**
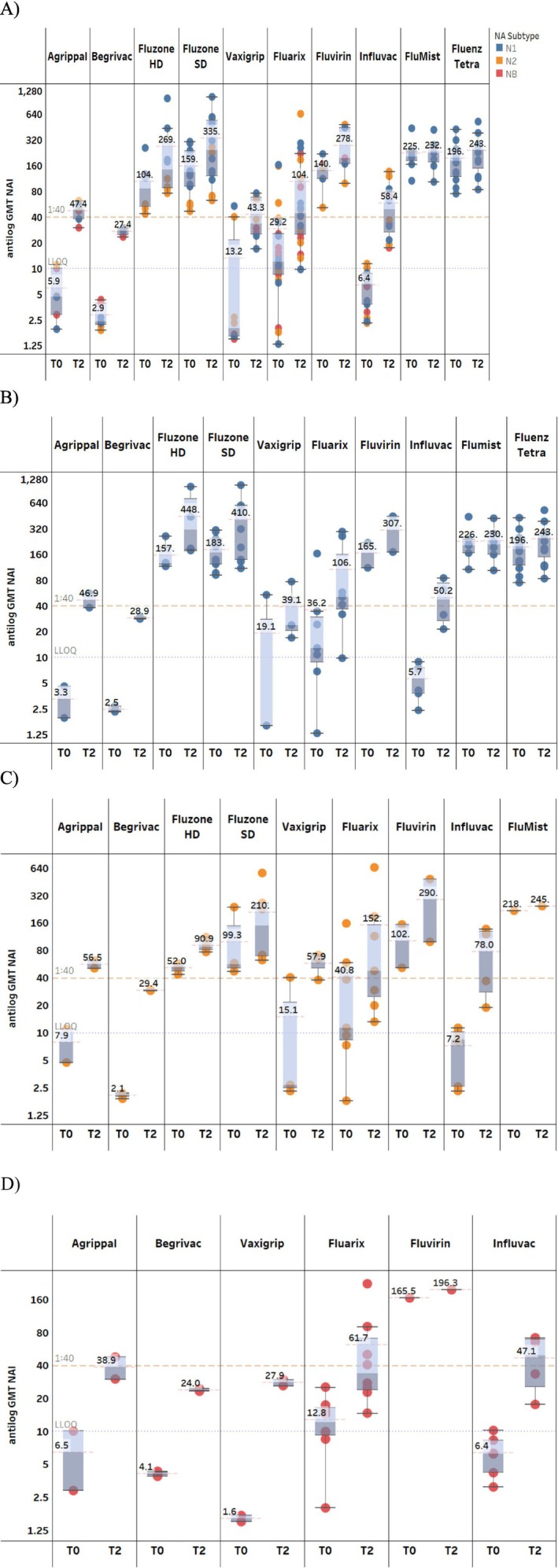
Post‐vaccination NAI antibody GMTs with various commercial influenza vaccines (including split‐virus, subunit, and live‐attenuated vaccines); studies that only had one data point contributing to the analysis were excluded. (A) All NA (sub)types (N1, N2, and influenza B virus NAs [NB]), (B) N1, (C) N2, and (D) NB. Data are summarized as box‐and‐whisker plots, where the boxes indicate the middle 50% of the data (i.e., middle two quartiles of the data's distribution) with whiskers to display all points within 1.5 times the interquartile range, and overlayed with the individual study data points included (each dot indicates unique data from the publications, and each color indicates a unique influenza A subtype or influenza B virus lineage). The bold numbers for each pane indicate the aggregated GMTs (dashed red line) for a particular time point within each commercial vaccine. The dashed orange line indicates 1:40 titer (assumed seroprotection level) and the dotted purple line indicates 1:10 titer (LLOQ). T0 = D0; T2 = D8–30. D, day; GMT, geometric mean titer; LLOQ, lower limit of quantification; NA, neuraminidase; NAI, neuraminidase inhibition; NB, influenza B virus NAs; T, time point.

All commercial influenza vaccines (including subunit and live‐attenuated formulations) demonstrated elevated NAI antibody GMTs against all three NA (sub)types from pre‐vaccination levels to T2 (days 8–30 post‐vaccination) (Figure 5). The magnitude of fold rise, however, appeared constrained by pre‐existing NAI antibody titers in several instances; notably, studies evaluating Fluenz Tetra and FluMist vaccines reported high baseline (T0) titers with minimal post‐vaccination increase (Figure S2).

Baseline NAI titers exhibited considerable heterogeneity in studies assessing different vaccine products. Studies assessing Agrippal, Begrivac, Influvac, and Vaxigrip demonstrated substantially lower baseline titers compared to other vaccines. This observation may be attributed to the study populations, which comprised individuals with potentially compromised immunity, including subjects with allergic bronchial asthma, hemodialysis recipients, and nursing home residents.

**FIGURE 6:**
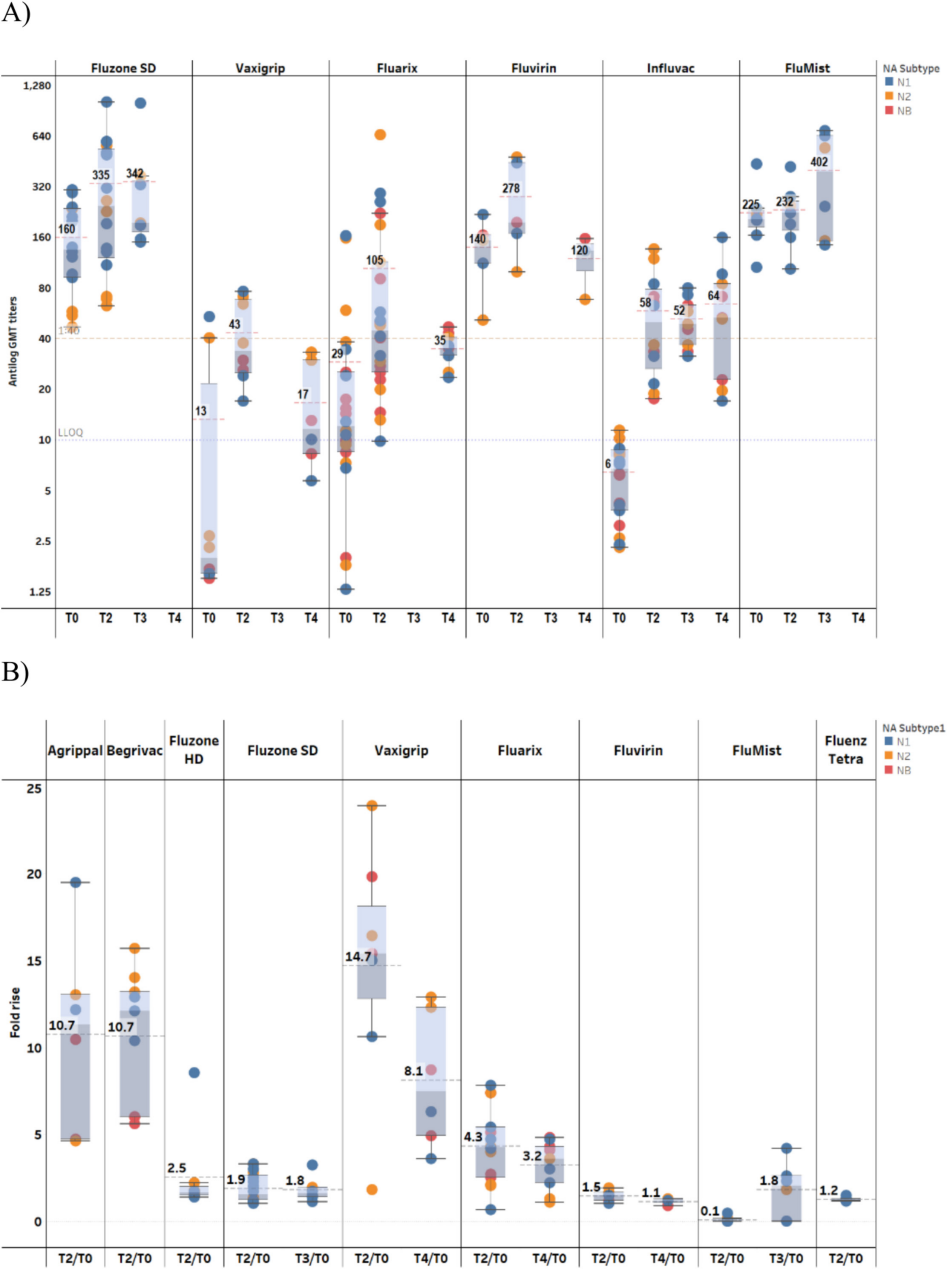
Durability of NAI response through to T4 A) and corresponding fold‐rise B). Data are summarized as box‐and‐whisker plots, where the boxes indicate the middle 50% of the data (i.e., middle two quartiles of the data's distribution) with whiskers to display all points within 1.5 times the interquartile range, and overlayed with the individual study data points included (each dot indicates a unique publication, and each color indicates a unique influenza A subtype or influenza B virus lineage). The bold numbers for each pane indicate the aggregated GMT (dashed red line) (or GMT fold‐rise [B]) for a particular time point within each commercial vaccine. The dashed orange line indicates 1:40 titer (assumed seroprotection level) and the dotted purple line indicates 1:10 titer (LLOQ). T0 = D0; T2 = D8–30; T4 = D91–180. D, day; GMT, geometric mean titer; LLOQ, lower limit of quantification; NAI, neuraminidase inhibition; T, time point.

Figure 6 illustrates the persistence of NAI antibody responses. NAI GMTs remained substantially elevated above baseline levels for all evaluated vaccine products through T4 (days 91–180 post‐vaccination), where follow‐up data were available, indicating sustained neuraminidase‐specific immunological responses following vaccination.

## 4 Discussion

This is the first systematic review to examine pre‐existing NAI antibody titers in adults as well as the induction and durability of these antibodies following vaccination with influenza vaccines. We found that pre‐existing NAI antibody titers were boosted following vaccination and remained at titers above pre‐vaccination levels up to 6 months after vaccination with split‐virus vaccines. Our analysis revealed important age‐related patterns in neuraminidase inhibition (NAI) antibody responses. For N1 subtype, no significant differences were observed between adults (18–64 years) and elderly (≥ 65 years) at any timepoint (*p* > 0.05). In contrast, adults generated significantly higher NAI antibody titers against N2 (*p* = 0.04) and NB subtypes (*p* = 0.01) during early post‐vaccination (days 8–30), as shown in Figure 4. We also found that high pre‐existing NAI antibody titers appear to limit the magnitude of the fold rise following vaccination. In addition, we found wide variation in NAI titers induced between the different commercial influenza vaccine types (including split‐virus, subunit, and live‐attenuated vaccines). Overall, our review provides insight on available evidence regarding NAI antibody responses in adults, which is increasingly being recognized as an independent correlate of protection [8, 11].

An NAI titer of 40 was shown to correspond to a vaccine efficacy of 55%–60% against influenza disease [69]. The NAI antibody GMTs achieved post‐vaccination (D8–30) with most of the vaccines assessed in this review were generally higher than a titer of 1:40 (Figure 5). This is encouraging, because the existing unstandardized NA content in licensed influenza vaccines may be contributing to some level of protection in addition to HAI responses. Therefore, new vaccines that include NA as an antigen are likely to provide more consistent protective benefit and necessitate the determination of the NA content in vaccines to provide optimal NAI responses in clinical development [70].

Previous studies that centered on HA‐specific antibody responses revealed that those with high pre‐existing HAI antibody titers also have reduced boosting (as determined by fold rise) with vaccination than those with lower titers [71, 72]. We found a similar effect on NAI antibody boosting with split‐virus influenza vaccines. A similar inverse relationship between fold rise of NAI antibody responses and pre‐existing titers was also observed following natural influenza infection [73]. Nonetheless, we found that subsequent vaccinations increased the NAI antibody titers and contributed to the durability of the responses.

The mechanism by which pre‐existing strain‐specific (HAI or NAI) antibodies limits subsequent vaccine responses is not fully understood. Several hypotheses have been proposed to explain this phenomenon. One prominent theory is epitope masking, in which pre‐existing antibodies bind to and conceal specific epitopes on the antigen. This binding prevents B cells from recognizing and binding to these epitopes or nearby regions, thereby inhibiting their activation and subsequent stimulation [66, 74]. Additionally, pre‐existing antibodies may accelerate the clearance of the vaccine antigen through Fc‐dependent mechanisms, reducing the amount of antigen available to prime new B cell responses [67]. Another possibility is that antigen–antibody complexes formed by pre‐existing antibodies may inhibit B cell activation through Fcγ receptor‐mediated mechanisms [74]. Understanding these mechanisms is crucial for improving vaccine design and efficacy, particularly in the context of developing vaccines that can elicit robust responses despite the presence of pre‐existing immunity.

Although immunosenescence is a plausible explanation for limited vaccine responses in the elderly, we observed minimal difference between adults aged 18–64 years and the elderly (≥ 65 years) in pre‐vaccination and post‐vaccination NAI antibody titers. In contrast, age was shown to strongly affect HAI antibody responses following vaccination with influenza vaccines, with the highest responses observed in the youngest adult age groups [72]. The main caveat with our age comparisons was that not all data (adults vs. elderly) compared were fully paired within the same publication but included cross‐publication comparisons, which may have limited identification of any differences.

Our review identifies certain gaps in the literature beyond the lack of systematic reporting of anti‐NA responses in vaccine trials. First, studies reporting anti‐NA antibodies did not provide any information on the amount of NA in the study vaccines. Second, there was limited data on the durability of the anti‐NA antibodies. Third, data from children and adolescents were generally lacking. Fourth, systematic reporting of anti‐NA response following natural infection is scarce. We found three publications [35, 41, 51] in our systematic review reporting anti‐NA responses after natural infection among a larger body of literature on the topic. One publication reported natural infection with influenza A virus elicited higher antibody affinity and neuraminidase inhibition (NAI) activity than after vaccination [51]. While we show durability of anti‐NA response up to 90 days after vaccination, there are conflicting reports on the topic after natural infection. Anti‐NA antibodies are found up to 4 years after infection vaccination while another showed a decline of anti‐NA within 5 months of infection [68, 75]. The head‐to‐head comparison of quality, quantity and durability of anti‐NA responses in vaccinees compared to natural infection remains to be more thoroughly studied.

The focus in vaccine development has evolved in recent years, from exclusively aiming to achieve neutralizing protection to now embracing and licensing vaccines that modulate disease. This paradigm shift in the vaccine landscape highlights the critical role of NA in future vaccine strategies. By reducing the severity of infections, these new vaccines not only enhance overall public health outcomes but also pave the way for broader inclusion of NA, underscoring the importance of this innovative approach.

This review has some limitations. There was a lack of standardization for the time points at which immunogenicity assessments were conducted, which necessitated combining data across a range of time points. The studies included encompassed a diverse range of participants and had wide variability in pre‐existing NAI antibody titers and thus wide variability in subsequent post‐vaccination responses. Moreover, only two studies [40, 41] assessed different age groups, making robust comparisons across age groups difficult. Thus, it is unclear whether the variability in pre‐ and post‐vaccination NAI antibody titers could be attributed to inherent variations in exposure to influenza viruses between seasons, the lack of NA standardization across the influenza vaccines, or assay variability/changes over time. There was a striking lack of publications that reported NAI antibody titers at time points > 30 days to allow for a robust assessment of durability, highlighting a significant gap in the NA literature. We also restricted our review and meta‐analysis to analyzing absolute antibody titers as an endpoint while other endpoints such as seroconversion rate and seroprotection rates have been reported. In addition, the studies included were mainly limited to those reporting homologous boosting of pre‐existing NAI antibodies. Future studies would be needed to address the role of pre‐existing NAI immunity on heterologous or heterosubtypic boosting. While enzyme‐linked immunosorbent assay (ELISA) may be considered a viable alternative to enzyme‐linked lectin assay (ELLA) [76], our review found insufficient studies reporting ELISA data for meaningful analysis. ELISA offers the advantage of high‐throughput sample processing. However, it primarily indicates antibody induction without necessarily reflecting functional or protective capacity. Nonetheless, previous research has demonstrated that antibody levels measured by ELISA correlate significantly with functional neuraminidase inhibition titers [40], suggesting its potential value in future investigations of vaccine‐induced neuraminidase‐specific immunity. Finally, there were fewer studies assessing antibodies to both influenza virus B lineages than those for the influenza virus A subtypes N1 and N2.

## 5 Conclusion

In conclusion, our review shows that all split‐virus influenza vaccines boost pre‐existing NAI antibody titers in adults. This review supports the need to better define and target NA content in influenza vaccines and perform routine assessment of NAI antibody responses in addition to HAI responses in clinical trials. As of 2025, we have created a database of reported NAI titers post‐vaccination to help inform future vaccine clinical trials targeting NA‐specific antibody responses. This database can easily be updated periodically as new information becomes available, which will enable us to better understand the benefits of NA‐induced immunity in protection against influenza, particularly with vaccine types other than split‐virus (including emerging platforms) where sufficient data were lacking.

## Author Contributions

**Vardhini Ganesh:** conceptualization, data curation, writing – original draft, writing – review and editing. **Justin Iampietro:** conceptualization, data curation, writing – original draft, writing – review and editing. **Saranya Sridhar:** conceptualization, data curation, writing – original draft, writing – review and editing. **Ana P. Goncalvez:** conceptualization, data curation, writing – original draft, writing – review and editing.

## Conflicts of Interest

All authors are employees of Sanofi and may hold shares and/or stock options in the company.

## Supporting information


**Table S1:** Search string for clinicaltrials.gov.
**Table S2:** Search string for Cochrane Library.
**Table S3:** Search string for Embase.
**Table S4:** Search string for PubMed.
**Table S5:** Search string for Trialtrove.
**Figure S1:** Risk of bias assessment of A) randomized controlled trails assessment using RoB 2 tool and B) nonrandomized controlled trials using ROBINS‐I tool for the studies that assessed split‐virus vaccines included in the data synthesis.
**Figure S2:** Summary of NAI GMT changes from baseline (T0) to T2 by study. Each color indicates a unique publication with the individual dots per publication indicating different treatment groups within the publication. The dashed orange line indicates 1:40 titer (assumed seroprotection level) and the dotted purple line indicates 1:10 titer (LLOQ). T0 = D0; T2 = D8–30. D, day; GMT, geometric mean titer; LLOQ, lower limit of quantification; NAI, neuraminidase inhibiting; T, time point.

## Data Availability

All data generated or analyzed during this study is included in the published article (and its supporting information files).

## References

[1] F. Krammer , G. J. D. Smith , R. A. M. Fouchier , et al., “Influenza,” Nature Reviews Disease Primers 4, no. 1 (2018): 3. 10.1038/s41572-018-0002-y PMC7097467 29955068

[2] World Health Organization , “Global Influenza Programme – Burden of Disease,” accessed July 13, 2022, https://www.who.int/teams/global‐influenza‐programme/surveillance‐and‐monitoring/burden‐of‐disease.

[3] W. Putri , D. J. Muscatello , M. S. Stockwell , and A. T. Newall , “Economic Burden of Seasonal Influenza in the United States,” Vaccine 36, no. 27 (2018): 3960–3966. 29801998 10.1016/j.vaccine.2018.05.057

[4] R. G. Webster , W. J. Bean , O. T. Gorman , T. M. Chambers , and Y. Kawaoka , “Evolution and Ecology of Influenza A Viruses,” Microbiological Reviews 56, no. 1 (1992): 152–179. 1579108 10.1128/mr.56.1.152-179.1992 PMC372859

[5] T. J. Wohlbold and F. Krammer , “In the Shadow of Hemagglutinin: A Growing Interest in Influenza Viral Neuraminidase and Its Role as a Vaccine Antigen,” Viruses 6, no. 6 (2014): 2465–2494. 24960271 10.3390/v6062465 PMC4074938

[6] S. Creytens , M. N. Pascha , M. Ballegeer , X. Saelens , and C. A. M. de Haan , “Influenza Neuraminidase Characteristics and Potential as a Vaccine Target,” Frontiers in Immunology 12 (2021): 786617. 34868073 10.3389/fimmu.2021.786617 PMC8635103

[7] C. I. Paules , H. D. Marston , R. W. Eisinger , D. Baltimore , and A. S. Fauci , “The Pathway to a Universal Influenza Vaccine,” Immunity 47, no. 4 (2017): 599–603. 29045889 10.1016/j.immuni.2017.09.007

[8] Y. Q. Chen , T. J. Wohlbold , N. Y. Zheng , et al., “Influenza Infection in Humans Induces Broadly Cross‐Reactive and Protective Neuraminidase‐Reactive Antibodies,” Cell 173, no. 2 (2018): 417–429.e410. 29625056 10.1016/j.cell.2018.03.030 PMC5890936

[9] K. B. Westgeest , M. de Graaf , M. Fourment , et al., “Genetic Evolution of the Neuraminidase of Influenza A (H3N2) Viruses From 1968 to 2009 and Its Correspondence to Haemagglutinin Evolution,” Journal of General Virology 93, no. Pt 9 (2012): 1996–2007. 22718569 10.1099/vir.0.043059-0 PMC3542130

[10] M. Rajendran , F. Krammer , and M. McMahon , “The Human Antibody Response to the Influenza Virus Neuraminidase Following Infection or Vaccination,” Vaccines (Basel) 9, no. 8 (2021): 846. 34451971 10.3390/vaccines9080846 PMC8402431

[11] A. S. Monto , J. G. Petrie , R. T. Cross , et al., “Antibody to Influenza Virus Neuraminidase: An Independent Correlate of Protection,” Journal of Infectious Diseases 212, no. 8 (2015): 1191–1199. 25858957 10.1093/infdis/jiv195

[12] G. Cortés , I. Ustyugova , T. Farrell , et al., “Boosting Neuraminidase Immunity in the Presence of Hemagglutinin With the Next Generation of Influenza Vaccines,” npj Vaccines 9, no. 1 (2024): 228. 39562599 10.1038/s41541-024-01011-x PMC11577023

[13] M. Hatta , Y. Hatta , A. Choi , et al., “An Influenza mRNA Vaccine Protects Ferrets From Lethal Infection With Highly Pathogenic Avian Influenza A(H5N1) Virus,” Science Translational Medicine 16, no. 778 (2024): eads1273. 39693411 10.1126/scitranslmed.ads1273 PMC12100637

[14] D. Moher , A. Liberati , J. Tetzlaff , D. G. Altman , and PRISMA Group , “Preferred Reporting Items for Systematic Reviews and Meta‐Analyses: The PRISMA Statement,” PLoS Medicine 6, no. 7 (2009): e1000097. 19621072 10.1371/journal.pmed.1000097 PMC2707599

[15] W. S. Richardson , M. C. Wilson , J. Nishikawa , and R. S. Hayward , “The Well‐Built Clinical Question: A Key to Evidence‐Based Decisions,” ACP Journal Club 123, no. 3 (1995): A12–A13. 10.7326/ACPJC-1995-123-3-A12 7582737

[16] J. A. C. Sterne , J. Savović , M. J. Page , et al., “RoB 2: A Revised Tool for Assessing Risk of Bias in Randomised Trials,” BMJ (Clinical research ed.) 366 (2019): l4898. 10.1136/bmj.l4898 31462531

[17] J. A. Sterne , M. A. Hernan , B. C. Reeves , et al., “ROBINS‐I: A Tool for Assessing Risk of Bias in Non‐Randomised Studies of Interventions,” BMJ (Clinical research ed.) 355 (2016): i4919. 10.1136/bmj.i4919 PMC5062054 27733354

[18] L. A. McGuinness and J. P. T. Higgins , “Risk‐Of‐Bias VISualization (ROBVIS): An R Package and Shiny Web App for Visualizing Risk‐of‐Bias Assessments,” Research Synthesis Methods 12, no. 1 (2021): 55–61. 32336025 10.1002/jrsm.1411

[19] R. B. Couch , R. L. Atmar , W. A. Keitel , et al., “Randomized Comparative Study of the Serum Antihemagglutinin and Antineuraminidase Antibody Responses to Six Licensed Trivalent Influenza Vaccines,” Vaccine 31, no. 1 (2012): 190–195. 23107591 10.1016/j.vaccine.2012.10.065 PMC3520601

[20] D. Bethell , D. Saunders , A. Jongkaewwattana , et al., “Evaluation of In Vitro Cross‐Reactivity to Avian H5N1 and Pandemic H1N1 2009 Influenza Following Prime Boost Regimens of Seasonal Influenza Vaccination in Healthy Human Subjects: A Randomised Trial,” PLoS ONE 8, no. 3 (2013): e59674. 23555741 10.1371/journal.pone.0059674 PMC3608534

[21] A. Mastalerz‐Migas , A. Steciwko , and L. B. Brydak , “Immune Response to Influenza Vaccine in Hemodialysis Patients With Chronic Renal Failure,” Advances in Experimental Medicine and Biology 756 (2013): 285–290. 22836646 10.1007/978-94-007-4549-0_35

[22] W. Zhong and M. Z. Levine , “Stockpiled Avian Influenza A(H7N9) Vaccines Induce Robust, Nonneutralizing Functional Antibodies Against Antigenically Drifted Fifth‐Wave A(H7N9) Viruses,” Journal of Infectious Diseases 220, no. 8 (2019): 1276–1280. 31169293 10.1093/infdis/jiz295

[23] L. B. Brydak , S. Tadeusz , and M. Magdalena , “Antibody Response to Influenza Vaccination in Healthy Adults,” Viral Immunology 17, no. 4 (2004): 609–615. 15671759 10.1089/vim.2004.17.609

[24] L. B. Brydak , M. Machala , J. Mysliwska , A. Mysliwski , and P. Trzonkowski , “Immune Response to Influenza Vaccination in an Elderly Population,” Journal of Clinical Immunology 23, no. 3 (2003): 214–222. 12797543 10.1023/a:1023314029788

[25] L. B. Brydak and M. Calbecka , “Immunogenicity of Influenza Vaccine in Patients With Hemato‐Oncological Disorders,” Leukemia & Lymphoma 32, no. 3–4 (1999): 369–374. 10037036 10.3109/10428199909167399

[26] E. D. Kilbourne , R. B. Couch , J. A. Kasel , et al., “Purified Influenza A Virus N2 Neuraminidase Vaccine Is Immunogenic and Non‐Toxic in Humans,” Vaccine 13, no. 18 (1995): 1799–1803. 8701596 10.1016/0264-410x(95)00127-m

[27] D. E. Sarateanu , W. Ehrengut , K. Pressler , M. Peukert , and K. D. Schenk , “Serological Response to Whole, Split and Subunit Influenza Vaccines of Persons With and Without Immunological Experience Towards Influenza A/U.S.S.R. 90/77 Virus,” Comparative Immunology, Microbiology and Infectious Diseases 3, no. 1–2 (1980): 225–236. 7471712 10.1016/0147-9571(80)90061-2

[28] K. Pressler , M. Peukert , D. Schenk , and M. Borgono , “Comparison of the Antigenicity and Tolerance of an Influenza Aluminum‐Oxide Adsorbate Vaccine With an Aqueous Vaccine,” Pharmatherapeutica 3, no. 3 (1982): 195–200. 7134227

[29] E. K. Kuwert , P. G. Höher , J. Werner , et al., “Neuraminidase Antibodies in Serum and Nasal Washings After Immunization by Means of Live and Killed Whole Virion, Split Virion and Subunit (HA and N) Influenza Vaccines,” Developments in Biological Standardization 39 (1977): 77–83. 604137

[30] G. R. Noble , H. S. Kaye , R. J. O'Brien , et al., “Persistence of Influenza A/New Jersey/76 (Hsw1N1) Antibody One Year After Vaccination,” Developments in Biological Standardization 39 (1977): 253–260. 604107

[31] C. W. Potter , R. Jennings , and A. Clark , “The Antibody Response and Immunity to Challenge Infection Induced by Whole, Inactivated and Tween‐Ether Split Influenza Vaccines,” Developments in Biological Standardization 39 (1977): 323–328. 604115

[32] S. Esposito , J. Nauta , G. Lapini , E. Montomoli , and S. van de Witte , “Efficacy and Safety of a Quadrivalent Influenza Vaccine in Children Aged 6‐35 Months: A Global, Multiseasonal, Controlled, Randomized Phase III Study,” Vaccine 40, no. 18 (2022): 2626–2634. 35315323 10.1016/j.vaccine.2022.02.088

[33] C. W. Potter , A. Clark , R. Jennings , G. C. Schild , J. M. Wood , and P. K. McWilliams , “Reactogenicity and Immunogenicity of Inactivated Influenza A (H1N1) Virus Vaccine in Unprimed Children. Report to the Medical Research Council Committee on Influenza and Other Respiratory Virus Vaccines,” Journal of Biological Standardization 8, no. 1 (1980): 35–48. 6995458 10.1016/s0092-1157(80)80045-x

[34] K. Jahnz‐Rózyk , L. B. Brydak , T. Targowski , M. Machala , and T. Plusa , “Effect of Influenza Vaccinations on Immune Response and Serum Eotaxin Level in Patients With Allergic Bronchial Asthma,” Mediators of Inflammation 13, no. 3 (2004): 195–199. 15223611 10.1080/09511920410001713501 PMC1781554

[35] J. G. Petrie , S. E. Ohmit , R. Truscon , et al., “Modest Waning of Influenza Vaccine Efficacy and Antibody Titers During the 2007‐2008 Influenza Season,” Journal of Infectious Diseases 214, no. 8 (2016): 1142–1149. 27095420 10.1093/infdis/jiw105

[36] D. C. Powers , E. D. Kilbourne , and B. E. Johansson , “Neuraminidase‐Specific Antibody Responses to Inactivated Influenza Virus Vaccine in Young and Elderly Adults,” Clinical and Diagnostic Laboratory Immunology 3, no. 5 (1996): 511–516. 8877127 10.1128/cdli.3.5.511-516.1996 PMC170398

[37] G. M. Air , J. Feng , T. Chen , M. L. Joachims , J. A. James , and L. F. Thompson , “Individual Antibody and T Cell Responses to Vaccination and Infection With the 2009 Pandemic Swine‐Origin H1N1 Influenza Virus,” Journal of Clinical Immunology 31, no. 5 (2011): 900–912. 21732013 10.1007/s10875-011-9563-1 PMC3197711

[38] L. B. Brydak , M. Romanowska , I. Nowak , A. Ciszewski , and Z. T. Bilinska , “Antibody Response to Influenza Vaccine in Coronary Artery Disease: A Substudy of the FLUCAD Study,” Medical Science Monitor 15, no. 7 (2009): PH85–PH91. 19564837

[39] N. G. Rouphael , L. Lai , S. Tandon , et al., “Immunologic Mechanisms of Seasonal Influenza Vaccination Administered by Microneedle Patch From a Randomized Phase I Trial,” npj Vaccines 6, no. 1 (2021): 89. 34262052 10.1038/s41541-021-00353-0 PMC8280206

[40] M. Rajendran , R. Nachbagauer , M. E. Ermler , et al., “Analysis of Anti‐Influenza Virus Neuraminidase Antibodies in Children, Adults, and the Elderly by ELISA and Enzyme Inhibition: Evidence for Original Antigenic Sin,” MBio 8, no. 2 (2017): e02281–e02216. 28325769 10.1128/mBio.02281-16 PMC5362038

[41] G. Muhamed , E. Greenbaum , and Z. Zakay‐Rones , “Neuraminidase Antibody Response to Inactivated Influenza Virus Vaccine Following Intranasal and Intramuscular Vaccination,” Israel Medical Association Journal 8, no. 3 (2006): 155–158. 16599048

[42] Y. Desheva , T. Smolonogina , S. Donina , and L. Rudenko , “Study of Neuraminidase‐Inhibiting Antibodies in Clinical Trials of Live Influenza Vaccines,” Antibodies (Basel) 9, no. 2 (2020): 20. 32485797 10.3390/antib9020020 PMC7344733

[43] A. Ben‐Yehuda , A. Joseph , E. Zeira , et al., “Immunogenicity and Safety of a Novel Liposomal Influenza Subunit Vaccine (INFLUSOME‐VAC) in Young Adults,” Journal of Medical Virology 69, no. 4 (2003): 560–567. 12601765 10.1002/jmv.10345

[44] R. Fritz , N. Sabarth , S. Kiermayr , et al., “A Vero Cell‐Derived Whole‐Virus H5N1 Vaccine Effectively Induces Neuraminidase‐Inhibiting Antibodies,” Journal of Infectious Diseases 205, no. 1 (2012): 28–34. 22090447 10.1093/infdis/jir711

[45] L. B. Brydak , J. Bialek , H. Rudnicka , A. Denys , H. L. Regnery , and N. Cox , “Seroconversion Assessment in a Billeted Military Medical University Student Group After Anti‐Influenza Subunit Vaccinations in 1993/1994 in Poland,” Antiinfect Drugs Chemother 15 (1997): 13–16.

[46] Y. Desheva , I. Sychev , T. Smolonogina , et al., “Anti‐Neuraminidase Antibodies Against Pandemic A/H1N1 Influenza Viruses in Healthy and Influenza‐Infected Individuals,” PLoS ONE 13, no. 5 (2018): e0196771. 29742168 10.1371/journal.pone.0196771 PMC5942809

[47] R. Fritz , N. Hetzelt , R. Ilk , et al., “Neuraminidase‐Inhibiting Antibody Response to H5N1 Virus Vaccination in Chronically Ill and Immunocompromised Patients,” Open Forum Infectious Diseases 1, no. 2 (2014): ofu072. 25734142 10.1093/ofid/ofu072 PMC4281780

[48] L.‐J. Chang , Y. Meng , H. Janosczyk , V. Landolfi , and H. K. Talbot , “Safety and Immunogenicity of High‐Dose Quadrivalent Influenza Vaccine in Adults ≥ 65 Years of Age: A Phase 3 Randomized Clinical Trial,” Vaccine 37, no. 39 (2019): 5825–5834. 31431411 10.1016/j.vaccine.2019.08.016

[49] K. Boonnak , J. Dhitavat , N. Thantamnu , et al., “Immune Responses to Intradermal and Intramuscular Inactivated Influenza Vaccine Among Older Age Group,” Vaccine 35, no. 52 (2017): 7339–7346. 29157960 10.1016/j.vaccine.2017.10.106

[50] A. Ben‐Yehuda , A. Joseph , Y. Barenholz , et al., “Immunogenicity and Safety of a Novel IL‐2‐Supplemented Liposomal Influenza Vaccine (INFLUSOME‐VAC) in Nursing‐Home Residents,” Vaccine 21, no. 23 (2003): 3169–3178. 12804845 10.1016/s0264-410x(03)00251-2

[51] L. B. Brydak , E. Ordynska , B. Wasilewski , H. Rudnicka , H. Regnery , and N. Cox , “Immunogenicity of Trivalent Subunit Influenza Vaccine in Elderly People With Chronic Medical Conditions Vaccinated in 1993 in Poland,” Anti‐Infective Agents in Medicinal Chemistry 15, no. 1 (1997): 9–12.

[52] R. B. Couch , R. G. Douglas, Jr. , D. S. Fedson , and J. A. Kasel , “Correlated Studies of a Recombinant Influenza‐Virus Vaccine. 3. Protection Against Experimental Influenza in Man,” Journal of Infectious Diseases 124, no. 5 (1971): 473–480. 5000515 10.1093/infdis/124.5.473

[53] C. Claeys , V. Chandrasekaran , J. Garcia‐Sicilia , et al., “Anamnestic Immune Response and Safety of an Inactivated Quadrivalent Influenza Vaccine in Primed Versus Vaccine‐Naive Children,” Pediatric Infectious Disease Journal 38, no. 2 (2019): 203–210. 30325891 10.1097/INF.0000000000002217 PMC6344072

[54] T. Nolan , P. Izurieta , B. W. Lee , et al., “Heterologous Prime‐Boost Vaccination Using an AS03B‐Adjuvanted Influenza A(H5N1) Vaccine in Infants and Children < 3 Years of Age,” Journal of Infectious Diseases 210, no. 11 (2014): 1800–1810. 24973461 10.1093/infdis/jiu359 PMC4224137

[55] M. Romanowska , A. Banaszkiewicz , I. Nowak , A. Radzikowski , and L. B. Brydak , “Immunization Against Influenza During the 2005/2006 Epidemic Season and the Humoral Response in Children With Diagnosed Inflammatory Bowel Disease (IBD),” Medical Science Monitor 16, no. 9 (2010): CR433–CR439. 20802416

[56] T. C. Chow , K. R. Beutner , and P. L. Ogra , “Cell‐Mediated Immune Responses to the Hemagglutinin and Neuraminidase Antigens of Influenza A Virus After Immunization in Humans,” Infection and Immunity 25, no. 1 (1979): 103–109. 478632 10.1128/iai.25.1.103-109.1979 PMC414427

[57] K. R. Beutner , T. Chow , E. Rubi , J. Strussenberg , J. Clement , and P. L. Ogra , “Evaluation of a Neuraminidase‐Specific Influenza A Virus Vaccine in Children: Antibody Responses and Effects on Two Successive Outbreaks of Natural Infection,” Journal of Infectious Diseases 140, no. 6 (1979): 844–850. 396336 10.1093/infdis/140.6.844

[58] P. L. Ogra , T. Chow , K. R. Beutner , et al., “Clinical and Immunologic Evaluation of Neuraminidase‐Specific Influenza A Virus Vaccine in Humans,” Journal of Infectious Diseases 135, no. 4 (1977): 499–506. 856917 10.1093/infdis/135.4.499

[59] K. R. Beutner , C. Rizzone , S. DeMello , and P. L. Ogra , “Clinical Evaluation of Neuraminidase Monospecific (HEqN2) Recombinant Influenza Vaccine in Children,” Developments in Biological Standardization 33 (1976): 162–170. 782964

[60] K. Hoschler , S. Maharjan , H. Whitaker , et al., “Use of Traditional Serological Methods and Oral Fluids to Assess Immunogenicity in Children Aged 2‐16 Years After Successive Annual Vaccinations With LAIV,” Vaccine 38, no. 12 (2020): 2660–2670. 32070679 10.1016/j.vaccine.2020.02.024 PMC7054836

[61] K. Hoschler , J. Southern , C. Thompson , et al., “Responses to Live Attenuated Influenza Vaccine in Children Vaccinated Previously With Pandemrix (ASO3(B) Adjuvanted Pandemic A/H1N1pdm09),” Vaccine 36, no. 21 (2018): 3034–3040. 29680198 10.1016/j.vaccine.2018.04.017

[62] R. Rokicka‐Milewska , L. B. Brydak , M. Machala , and A. Klukowska , “Antibody Response to Influenza Vaccine in Children With Severe and Mild Hemophilia,” International Journal of Pediatric Hematology/Oncology 7, no. 1 (2000): 21–27.

[63] T. R. Cate , Y. Rayford , D. Niño , et al., “A High Dosage Influenza Vaccine Induced Significantly More Neuraminidase Antibody Than Standard Vaccine Among Elderly Subjects,” Vaccine 28, no. 9 (2010): 2076–2079. 20044052 10.1016/j.vaccine.2009.12.041 PMC2853016

[64] M. R. Laguio‐Vila , M. G. Thompson , S. Reynolds , et al., “Comparison of Serum Hemagglutinin and Neuraminidase Inhibition Antibodies After 2010‐2011 Trivalent Inactivated Influenza Vaccination in Healthcare Personnel,” Open Forum Infectious Diseases 2, no. 1 (2015): ofu115. 25884004 10.1093/ofid/ofu115 PMC4396428

[65] M. V. van der Velden , R. Fritz , E. M. Pöllabauer , et al., “Safety and Immunogenicity of a Vero Cell Culture‐Derived Whole‐Virus Influenza A(H5N1) Vaccine in a Pediatric Population,” Journal of Infectious Diseases 209, no. 1 (2014): 12–23. 24041789 10.1093/infdis/jit498

[66] C. E. Martina , J. E. Crowe, Jr. , and J. Meiler , “Glycan Masking in Vaccine Design: Targets, Immunogens and Applications,” Frontiers in Immunology 14 (2023): 1126034. 37033915 10.3389/fimmu.2023.1126034 PMC10076883

[67] T. Dangi , S. Sanchez , M. H. Lew , et al., “Pre‐Existing Immunity Modulates Responses to mRNA Boosters,” Cell Reports 42, no. 3 (2023): 112167. 36857186 10.1016/j.celrep.2023.112167 PMC9928730

[68] A. J. Smith and J. R. Davies , “Natural Infection With Influenza A (H3N2). The Development, Persistence and Effect of Antibodies to the Surface Antigens,” Epidemiology and Infection 77 (1976): 271–282. 10.1017/s0022172400024712 PMC2129857 185291

[69] P. B. Gilbert , Y. Fong , M. Juraska , et al., “HAI and NAI Titer Correlates of Inactivated and Live Attenuated Influenza Vaccine Efficacy,” BMC Infectious Diseases 19, no. 1 (2019): 453. 31117986 10.1186/s12879-019-4049-5 PMC6530189

[70] F. Krammer , R. A. M. Fouchier , M. C. Eichelberger , et al., “NAction! How Can Neuraminidase‐Based Immunity Contribute to Better Influenza Virus Vaccines?,” MBio 9, no. 2 (2018): e02332–e02317. 10.1128/mBio.02332-17 PMC5885027 29615508

[71] R. Pyhälä , V. Kumpulainen , S. Alanko , and T. Forsten , “HI Antibody Kinetics in Adult Volunteers Immunized Repeatedly With Inactivated Trivalent Influenza Vaccine in 1990‐1992,” Vaccine 12, no. 10 (1994): 947–952. 7975836 10.1016/0264-410x(94)90039-6

[72] T. A. Olafsdottir , K. F. Alexandersson , G. Sveinbjornsson , et al., “Age and Influenza‐Specific Pre‐Vaccination Antibodies Strongly Affect Influenza Vaccine Responses in the Icelandic Population Whereas Disease and Medication Have Small Effects,” Frontiers in Immunology 8 (2018): 1872. 29358933 10.3389/fimmu.2017.01872 PMC5766658

[73] H. M. T. K. Karunarathna , R. A. P. M. Perera , V. J. Fang , H. L. Yen , B. J. Cowling , and M. Peiris , “Serum Anti‐Neuraminidase Antibody Responses in Human Influenza A(H1N1)pdm09 Virus Infections,” Emerging Microbes & Infections 8, no. 1 (2019): 404–412. 30898033 10.1080/22221751.2019.1572433 PMC6455630

[74] V. I. Zarnitsyna , J. Lavine , A. Ellebedy , R. Ahmed , and R. Antia , “Multi‐Epitope Models Explain How Pre‐Existing Antibodies Affect the Generation of Broadly Protective Responses to Influenza,” PLoS Pathogens 12, no. 6 (2016): e1005692. 27336297 10.1371/journal.ppat.1005692 PMC4918916

[75] G. C. Schild , “Antibody Against Influenza A2 Virus Neuraminidase in Human Sera,” Journal of Hygiene 67 (1969): 353–365. 5256462 10.1017/s0022172400041759 PMC2130706

[76] M. C. Eichelberger and A. S. Monto , “Neuraminidase, the Forgotten Surface Antigen, Emerges as an Influenza Vaccine Target for Broadened Protection,” Journal of Infectious Diseases 219, no. Suppl 1 (2019): S75–S80. 30715357 10.1093/infdis/jiz017 PMC7325326

