# Supplementary material for: Human Immune Response to Influenza Neuraminidase After Vaccination: A Systematic Review
Source: Influenza Other Respir Viruses. 2025 Dec 9;19(12):e70192. doi: 10.1111/irv.70192 (PMC12981527; doi:10.1111/irv.70192)

**Supplementary Materials**

**Table S1:** Search string for clinicaltrials.gov.

| Search | Terms | Hits |
| --- | --- | --- |
| Condition or disease | Influenza | **45** |
| Other terms | Neuraminidase |  |
| Intervention/treatment | Vaccine |  |

**Table S2:** Search string for Cochrane Library.

| Search | Terms | Hits |
| --- | --- | --- |
| #1 | MeSH descriptor: [Neuraminidase] explode all trees and with qualifier(s): [antagonists & inhibitors – AI, immunology – IM] | 87 |
| #2 | MeSH descriptor: [Influenza, Human] explode all trees | 2,738 |
| #3 | MeSH descriptor: [Influenza Vaccines] explode all trees and with qualifier(s): [immunology – IM] | 895 |
| #4 | #2 OR #3 | 3,042 |
| #5 | MeSH descriptor: [Vaccines] explode all trees | 13,126 |
| #6 | MeSH descriptor: [Infections] explode all trees | 70,189 |
| #7 | MeSH descriptor: [Immunity] explode all trees | 4,024 |
| #8 | #5 OR #6 OR #7 | 77,947 |
| #9 | (“clinical study”):pt | 36 |
| #10 | (“clinical trial”):pt | 327,037 |
| #11 | #9 OR #10 | 327,072 |
| #12 | #8 AND #4 | 2,847 |
| #13 | #12 AND #11 | 1,106 |
| #14 | #13 AND #1 | **48** |

MeSH, Medical Subject Headings

**Table S3:** Search string for Embase.

| Search | Terms | Hits |
| --- | --- | --- |
| #1 | ‘flu’/exp OR ‘flu’ OR ‘flue’/exp OR ‘flue’ OR ‘Influenza’/exp OR ‘Influenza’ OR ‘Influenza Infection’/exp OR ‘Influenza Infection’ OR ‘Influenza, human’/exp OR ‘Influenza, human’ | 204,283 |
| #2 | ‘vaccine’/exp OR ‘combined vaccine’ OR ‘vaccin’ OR ‘vaccine’ OR ‘vaccine control’ OR ‘vaccine efficacy’ OR ‘vaccine potency’ OR ‘vaccine safety’ OR ‘vaccines’ OR ‘vaccines, combined’ OR ‘vaccines, conjugate’ | 497,560 |
| #3 | #1 and #2 | 60,129 |
| #4 | ‘placebo’/exp OR ‘Influenza vaccine’ | 416,601 |
| #5 | #3 and #4 | 43,074 |
| #6 | ‘sialidase’/exp OR ‘acylneuraminyl hydrolase’ OR ‘e.c. 3.2.1.18’ OR ‘mucopolysaccharide n acetylneuraminyl hydrolase’ OR ‘mucopolysaccharide n acetylneuraminylhydrolase’ OR ‘n acetyl neuraminate glycohydrolase’ OR ‘n acetylneuraminate glycohydrolase’ OR ‘neuraminidase’ OR ‘neuroaminidase’ OR ‘sialidase’ | 26,585 |
| #7 | #5 and #6 | 2,213 |
| #8 | ‘clinical trial’/exp OR ‘clinical drug trial’ OR ‘clinical trial’ OR ‘major clinical trial’ OR ‘trial, clinical’ OR ‘randomized controlled trial’/exp OR ‘observational study’/exp OR ‘observation studies’ OR ‘observation study’ OR ‘observational studies’ OR ‘observational studies as topic’ OR ‘observational study’ OR ‘observational study as topic’ | 2,196,482 |
| #9 | #7 and #8 | **883** |

**Table S4:** Search string for PubMed.

| Search | Terms | Hits |
| --- | --- | --- |
| #1 | ["Influenza, Human/epidemiology"[MeSH] OR "Influenza, Human/immunology"[MeSH] OR "Influenza, Human/mortality"[MeSH] OR "Influenza, Human/prevention and control"[MeSH] OR "Influenza, Human/statistics and numerical data"[MeSH] OR " Human Influenzas" OR "Influenza, Human" OR Influenza OR Influenzas OR "Human Flu" OR "Flu, Human" OR "Human influenza" OR "Influenza in Humans" OR “Influenza in Human" OR Grippe] | 140,031 |
| #2 | [vaccine OR vaccination OR immunization OR vaccines OR infection] | 4,429,414 |
| #3 | #1 AND #2 | 117,944 |
| #4 | [Clinical Study [Publication Type] OR Clinical trial [Publication Type] OR Randomized clinical trial [Publication Type]] | 990,363 |
| #5 | #3 AND #4 | 5,364 |
| #6 | neuraminidase or NAI or anti-NA | 25,862 |
| #7 | #5 AND #6 | **955** |

MeSH, Medical Subject Headings.

**Table S5:** Search string for Trialtrove.

| Terms | Hits |
| --- | --- |
| - Influenza as Disease/MeSH term/Patient segment **AND** - Neuraminidase as Primary endpoint/Other Endpoints/Target/Trial Objective **AND** - Vaccine or Immunization OR Vaccination OR Immunisation as Patient segment/Therapeutic area/Therapeutic class/Trial title/Trial objective/Treatment plan **AND** - Titers OR GMT OR Immune Response OR Antibody Response OR Humoral response OR Seroconversion OR Seroconvert OR Sero conversion OR Sero Convert as Trial title/Primary endpoint/Other Endpoints **AND NOT** - Neuraminidase inhibitor OR Tamiflu OR Oseltamivir as Trial Objective/Trial title/Mechanism of Action/Tested Drug | **26** |

MeSH, Medical Subject Headings

**Figure S1.** Risk of bias assessment of A) randomized controlled trails assessment using RoB 2 tool and B) non-randomized controlled trials using ROBINS-I tool for the studies that assessed split-virus vaccines included in the data synthesis.

A)


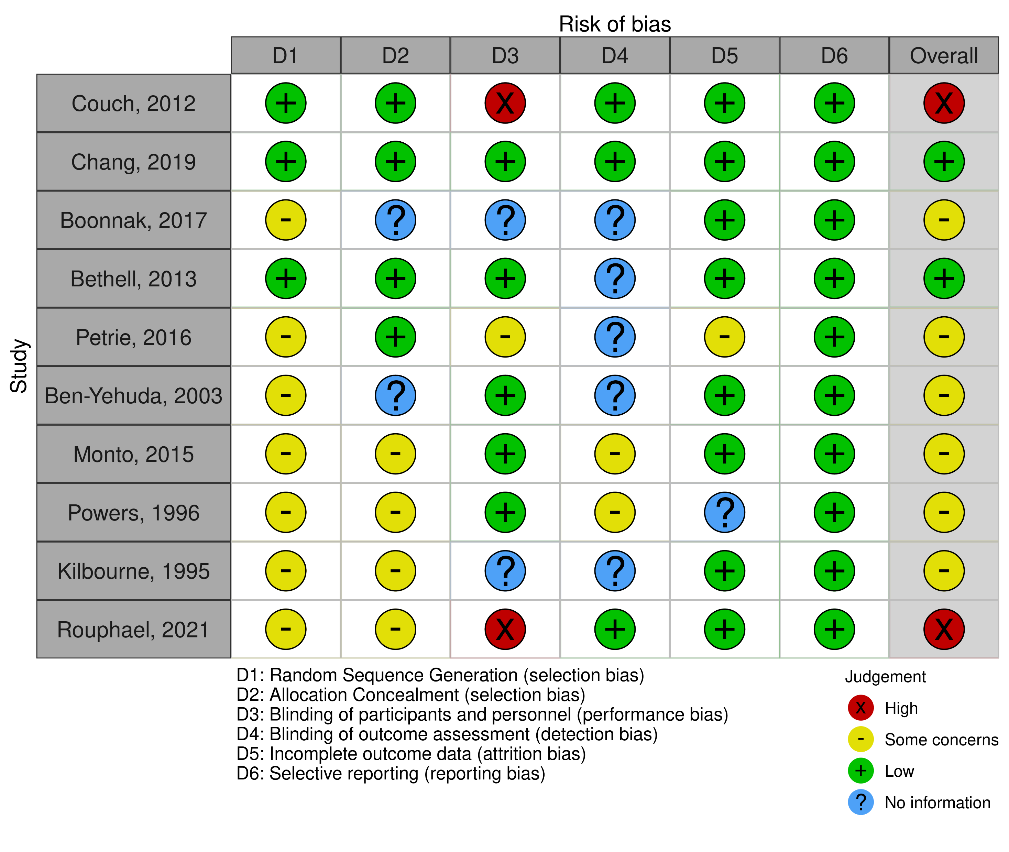


B)


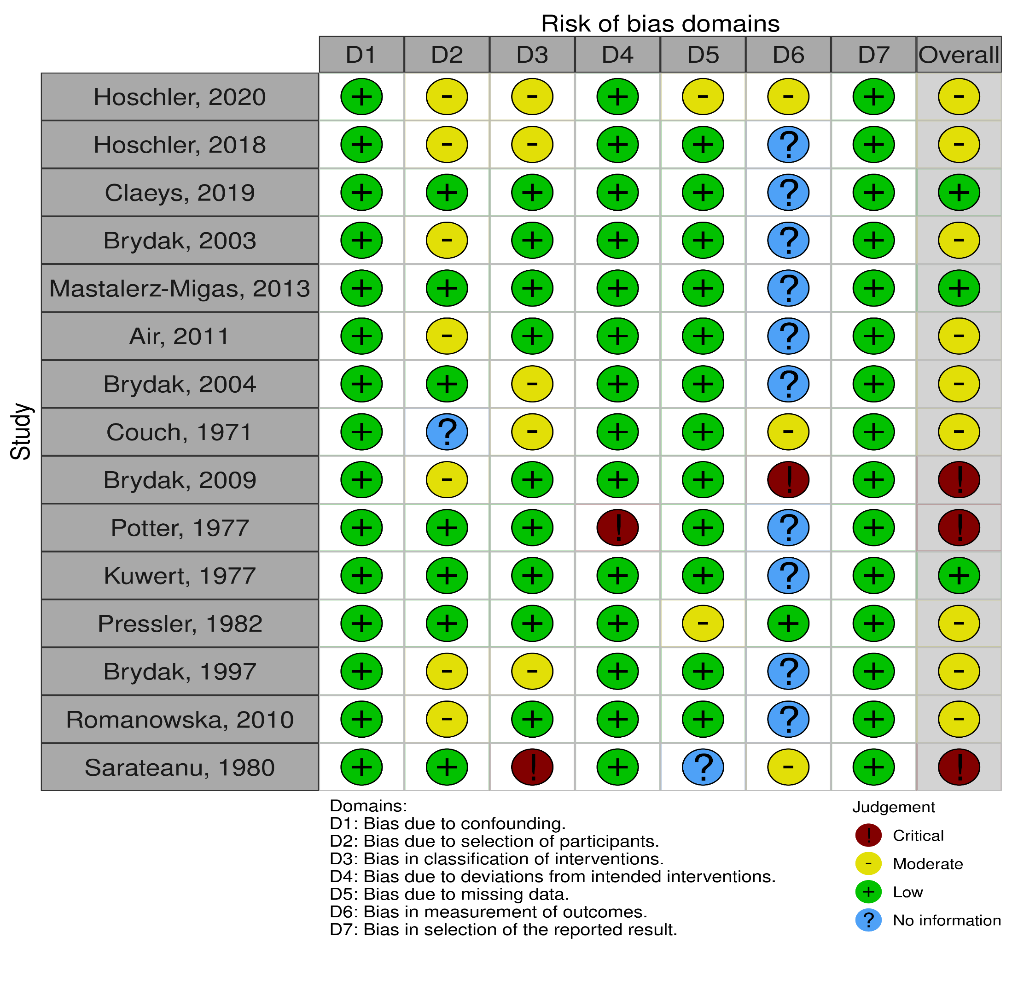


**Figure S2.** Summary of NAI GMT changes from baseline (T0) to T2 by study. Each color indicates a unique publication with the individual dots per publication indicating different treatment groups within the publication. The dashed orange line indicates 1:40 titer (assumed seroprotection level) and the dotted purple line indicates 1:10 titer (LLOQ). T0 = D0; T2 = D8–30. D, day; GMT, geometric mean titer; LLOQ, lower limit of quantification; NAI, neuraminidase inhibiting; T, time point.


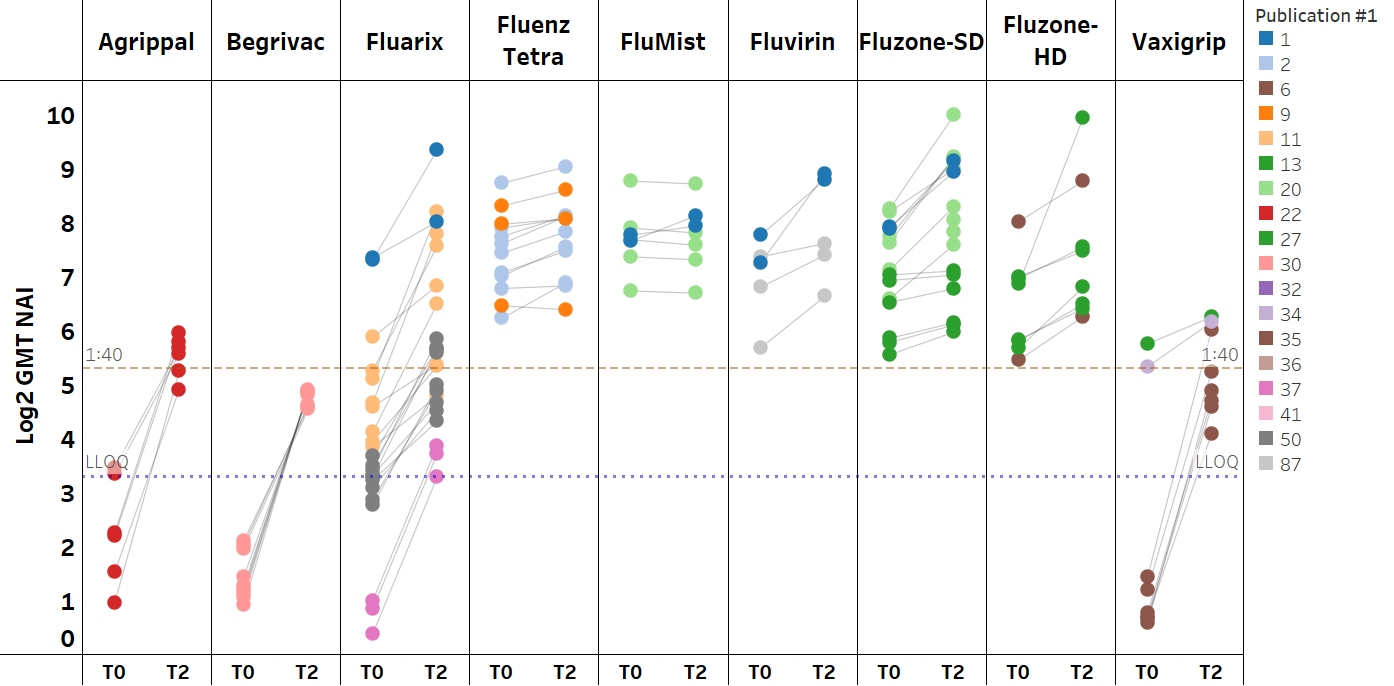

Supplement: Supplementary file 1 — Table S1: Search string for clinicaltrials.gov. Table S2: Search string for Cochrane Library. Table S3: Search string for Embase. Table S4: Search string for PubMed. Table S5: Search string for Trialtrove. Figure S1: Risk of bias assessment of A) randomized controlled trails assessment using RoB 2 tool and B) nonrandomized controlled trials using ROBINS‐I tool for the studies that assessed split‐virus vaccines included in the data synthesis. Figure S2: Summary of NAI GMT changes from baseline (T0) to T2 by study. Each color indicates a unique publication with the individual dots per publication indicating different treatment groups within the publication. The dashed orange line indicates 1:40 titer (assumed seroprotection level) and the dotted purple line indicates 1:10 titer (LLOQ). T0 = D0; T2 = D8–30. D, day; GMT, geometric mean titer; LLOQ, lower limit of quantification; NAI, neuraminidase inhibiting; T, time point. [file IRV-19-e70192-s001.docx]
